# EmergeNet: A novel deep-learning based ensemble segmentation model for emergence timing detection of coleoptile

**DOI:** 10.3389/fpls.2023.1084778

**Published:** 2023-02-03

**Authors:** Aankit Das, Sruti Das Choudhury, Amit Kumar Das, Ashok Samal, Tala Awada

**Affiliations:** ^1^ Institute of Radio Physics and Electronics, University of Calcutta, Kolkata, West Bengal, India; ^2^ School of Computing, University of Nebraska-Lincoln, Lincoln, NE, United States; ^3^ School of Natural Resources University of Nebraska-Lincoln, Lincoln, NE, United States; ^4^ Department of Computer Science and Engineering, Institute of Engineering and Management, Kolkata, West Bengal, India; ^5^ Agricultural Research Division, University of Nebraska-Lincoln, Lincoln, NE, United States

**Keywords:** event-based plant phenotyping, deep-learning, ensemble segmentation, emergence time detection, benchmark dataset

## Abstract

The emergence timing of a plant, i.e., the time at which the plant is first visible from the surface of the soil, is an important phenotypic event and is an indicator of the successful establishment and growth of a plant. The paper introduces a novel deep-learning based model called EmergeNet with a customized loss function that adapts to plant growth for coleoptile (a rigid plant tissue that encloses the first leaves of a seedling) emergence timing detection. It can also track its growth from a time-lapse sequence of images with cluttered backgrounds and extreme variations in illumination. EmergeNet is a novel ensemble segmentation model that integrates three different but promising networks, namely, SEResNet, InceptionV3, and VGG19, in the encoder part of its base model, which is the UNet model. EmergeNet can correctly detect the coleoptile at its first emergence when it is tiny and therefore barely visible on the soil surface. The performance of EmergeNet is evaluated using a benchmark dataset called the University of Nebraska-Lincoln Maize Emergence Dataset (UNL-MED). It contains top-view time-lapse images of maize coleoptiles starting before the occurrence of their emergence and continuing until they are about one inch tall. EmergeNet detects the emergence timing with 100% accuracy compared with human-annotated ground-truth. Furthermore, it significantly outperforms UNet by generating very high-quality segmented masks of the coleoptiles in both natural light and dark environmental conditions.

## 1 Introduction

Image-based plant phenotyping has the potential to transform the field of agriculture through the automated measurements of phenotypic expressions, i.e., observable biophysical traits of a plant as a result of complex interactions between genetics and environmental conditions. Accurate computation of meaningful phenotypes contributes to the study of high yield of better-quality crops with minimum resources (Das Choudhury et al., 2018). A plant’s phenome is defined as its observable characteristics or traits and is determined by the complex interaction between genotype and the environment. Plant phenotyping analysis has been an active research field for some time that adds to the understanding of yield and resource acquisition, and therefore, accelerates breedingcycles, improves our understanding of plant responses to environmental stresses, and contributes to global food security under changing climate. Image-based plant phenotypes can be broadly classified into three categories: structural, physiological, and event-based (Das Choudhury et al., 2019). The structural phenotypes characterize a plant’s morphology, whereas physiological phenotypes refer to the physiological processes that regulate plant growth and metabolism (Das Choudhury and Samal, 2020).

The timing detection of important events in a plant’s life cycle, for example, the emergence of coleoptile (i.e., protective sheath covering the emerging shoot) and new leaves, flowering, and fruiting, from time-lapse sequences has recently drawn significant research attention. Such phenotypes are called event-based phenotypes and provide crucial information in understanding the plant’s vigor, which varies with the interaction between genotype and environment. While interest in event-based phenotyping forleaves, flowers, and fruits has increased substantially in recent times (Wang et al., 2019; Bashyam et al., 2021), detecting the emergence and monitoring of the growth of the coleoptile based on computer vision and artificial intelligence techniques is a budding research field with vast opportunities for exploration. Emergence is a significant phenotype that not only helps determine the dormancy of seeds for different genotypes in different environmental conditions but also various aspects of early plant growth stages.

Unlike the visual tracking of rigid bodies, for instance, vehicles and pedestrians, the emergence timing detection of living organs and tracking their growth over time requires a different problem formulation with an entirely new set of challenges. Firstly, the state-of-the-art rigid body object detection and tracking methods deal with objects of considerably larger size that do not change in shape and appearance during the period of consideration. In contrast, our problem is to detect the coleoptile at emergence, when it is tiny in appearance, and track its dynamics as leaves emerge and grow into a seedling. The growth monitoring of size and shape is obtained as a by-product of an ensemble segmentation technique that segments the coleoptile with high accuracy. Secondly, the background (soil) in typical emergence detection in high-throughput plant phenotyping systems is significantly more complex than the state-of-the-art visual tracking applications. The soil substrate is multicolored due to the presence of perlite and vermiculite which makes the background cluttered, rendering the detection of a tiny coleoptile extremely challenging. Finally, the images are captured for a longer time than visual tracking, typically days, in a greenhouse with natural and artificial lighting conditions resulting in significant illumination variations.

The central contribution of this paper is to introduce a novel ensemble segmentation model tailored to the detection and growth monitoring of living organs in cluttered backgrounds and illumination variations for applications in event-based plant phenotyping. EmergeNet, characterized by its custom-designed loss function, uses a novel weighted ensemble learning technique to minimize the variance of the predicted masks and the generalization error for emergence timing detection. A benchmark dataset is indispensable for the development of the algorithm and performance comparison. Therefore, we have developed a publicly available benchmark dataset called the University of Nebraska-Lincoln Maize Emergence Dataset (UNL-MED) consisting of time-lapse image sequences of maize coleoptiles under the aforementioned conditions.

## 2 Related works

Multiple object tracking is challenging, yet it is of fundamental importance for many real-life practical applications (Xing et al., 2011). The survey paper by (Dhaka et al., 2021) provided comparisons of various convolutional neural networks and optimization techniques that are applied to predict plant diseases from leaf images. A comprehensive survey of multiple object tracking methods based on deep-learning is provided by (Xu et al., 2019). The method in (Xing et al., 2011) uses a progressive observation model followed by a dual-mode two-way Bayesian inference-based tracking strategy to track multiple highly interactive players with an abrupt view and pose variations in different sports videos, e.g., football, basketball, as well as hockey. A. Yilmaz et al (Yilmaz et al., 2006) showed that a plethora of research had been done in the field of object detection and tracking using various methods, including deep-learning algorithms. The method by (Aggarwal and Cai, 1999) gave an overview of the tasks involved in the motion analysis of a human body. (Dollár et al., 2009) worked on pedestrian detection, a key problem in computer vision, and proposed improved evaluation metrics. Computer vision based vehicle detection and tracking play an important role in the intelligent transport system (Min et al., 2018). The method in (Min et al., 2018) presents an improved ViBe for accurate detection of vehicles and uses two classifiers, i.e., support vector machine and convolutional neural network, to track vehicles in the presence of occlusions.

However, the use of deep neural networks for event-based plant phenotyping is in the early stage of research. The MangoYOLO algorithm (Wang et al., 2019) uses the YOLO object detector for detecting, tracking, and counting mangoes from a time-lapse video sequence. The method uses the Hungarian algorithm to correlate fruits between neighboring frames and a Kalman filter to predict the position of fruits in the following frames. A method for plant emergence detection and growth monitoring of the coleoptile based on adaptive hierarchical segmentation and optical flow using spatio-temporal image sequence analysis is presented in (Agarwal, 2017). A notable study in this domain includes the detection of budding and bifurcation events from 4D point clouds using a forward-backward analysis framework (Li et al., 2013). For a large-scale phenotypic experiment, the seeds are usually sown in smaller pots until germination and then transplanted to bigger pots based on a visual inspection of the germination date, size, and health of the seedlings. The method by (Scharr et al., 2020) developed an image-based automated germination detection system based on transfer-learning deep neural networks equipped with a visual support system for inspecting and transplanting seedlings. Deep-learning based ensemble segmentation technique has been recently introduced in medical image processing in ratio-based sampling for the arteries and veins in abdominal CT scans (Golla et al., 2020), skin lesion diagnosis using dermoscopic images (Arulmurugan et al., 2021), and portrait segmentation for application in surveillance systems (Kim et al., 2021).

To the best of our knowledge, there is no previous research that accurately detects the emergence timing of seedlings from a cluttered soil background and tracks its growth over a time-lapse sequence under extreme variations in illuminations using deep-learning based ensemble segmentation with custom loss functions. This paper proposes a novel algorithm that not only detects the emergence of the coleoptile under all the aforementioned challenging conditions but also successfully tracks its growth by creating an overlay mask on the image sequence even under extremely low light conditions at night. The proposed model, EmergeNet, uses deep-learning algorithms to create a novel segmentation model which can predict segmentation masks with high accuracy. We also release a benchmark dataset with ground-truth called UNL-MED, consisting of 3832 high-definition time-lapse image sequences of the maize coleoptiles.

## 3 Materials and methods

### 3.1 Dataset description

**Figure 1:**
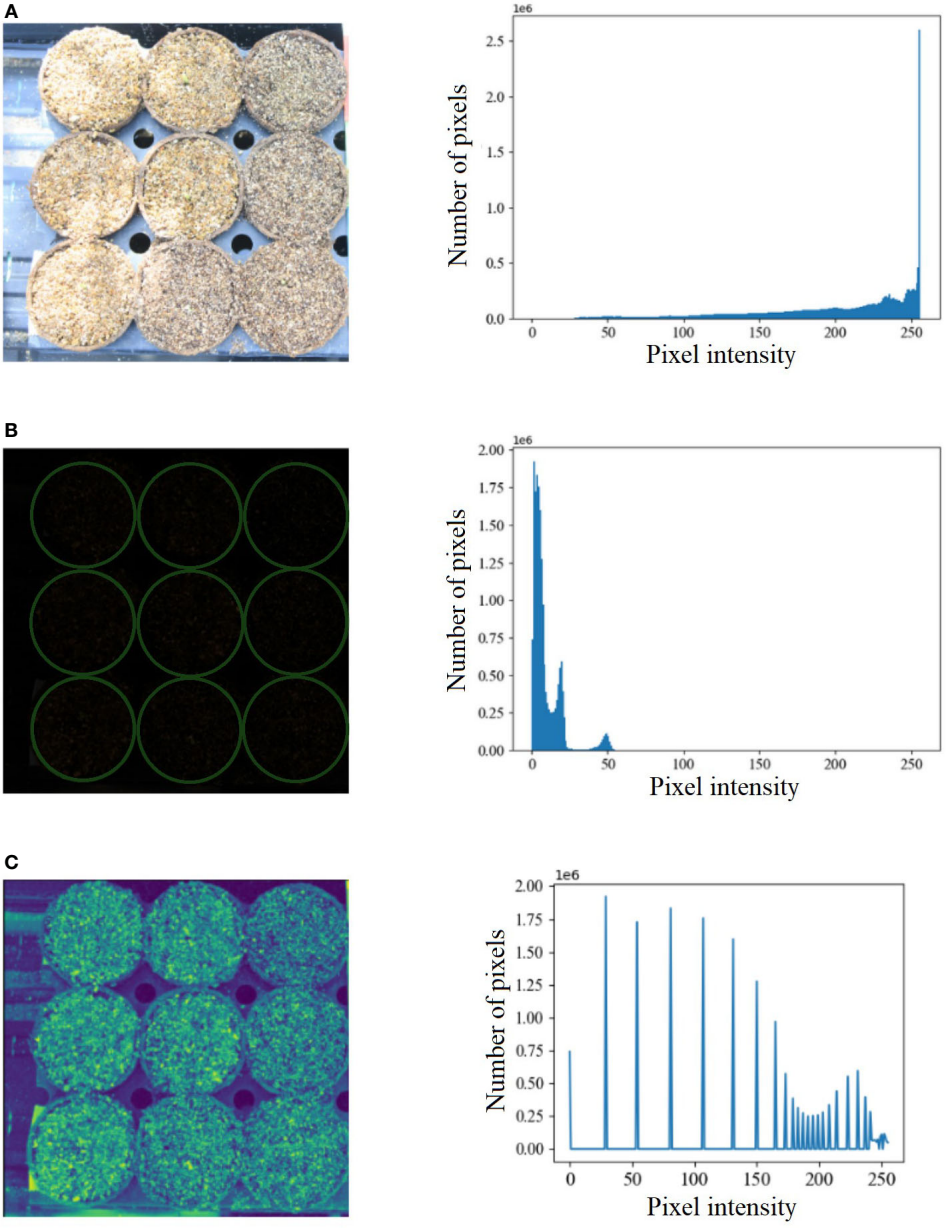
**(A)** An example of the brightest image from UNL-MED and its corresponding histogram; **(B)** an example of the darkest image from UNL-MED (the pots are marked in green circles) and its corresponding histogram; and **(C)** the darkest image after histogram equalization and its corresponding histogram.

Benchmark datasets are critical in developing new algorithms and performing uniform comparisons among state-of-the-art algorithms. Hence, we created a benchmark dataset called the UNL-MED. It is organized into two folders, namely, ‘Dataset’ and ‘Training’. The ‘Dataset’ folder contains all of the 3832 raw high-definition images of resolution 5184 × 3456. The ‘Training’ folder contains two subfolders, namely, ‘images’, which has randomly selected 130 images for training, and ‘masks’, which contains 130 corresponding masks but downsampled to a resolution of 256 × 256. The images are captured at an interval of two minutes under various external conditions, including varying illumination, cluttered background, warm and cool tone, starting from before the emergence occurred until the coleoptile is about 1 inch high to facilitate its growth monitoring. **Figure 1** is used to demonstrate an example of extreme contrast of illumination of the images used in the experiment based on histogram analysis. **Figure 1A** shows one of the brightest images and its histogram, whereas **Figure 1B** shows one of the darkest images and its corresponding histogram. **Figure 1C** shows the darkest image after histogram equalization and its histogram. Each image contains nine pots sowed with maize seeds of different genotypes. A visible light camera fitted with a tripod was placed directly above the nursery to capture high-definition top-view images of all nine pots every two minutes. The dataset can be freely downloaded from https://plantvision.unl.edu/dataset.

### 3.2 Dataset pre-processing

**Figure 2:**
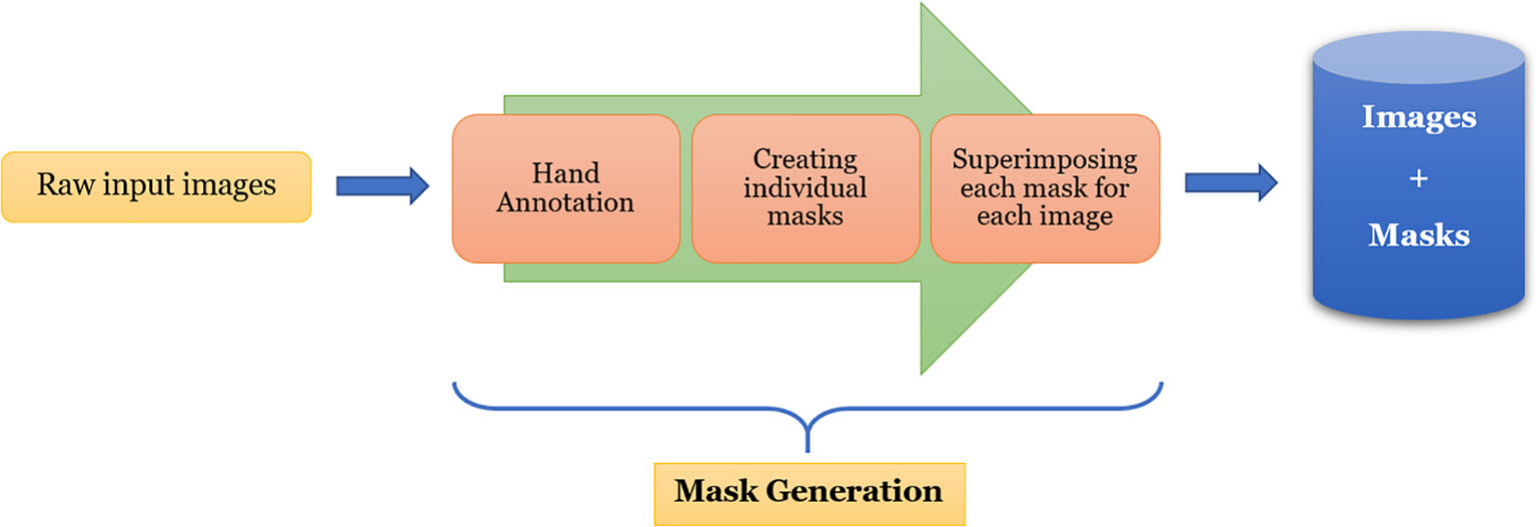
Illustration of mask generation process for UNL-MED.

One of the most challenging and tedious tasks in image segmentation using deep-learning is the generation of ground-truth. In our case, it is in the form of binary masks corresponding to the plants in the images. For a custom dataset like the one used in this work, it is imperative that the masks are generated accurately, and therefore, it needs to be done manually. Utmost care has been taken while generating these masks as these serve as monitoring information during the semantic segmentation training to provide feedback to the neural network. In our experiment, we made use of the open-source manual annotation software developed by Visual Geometry Group (VGG) (Dutta et al., 2016). The flowchart of the data pre-processing is shown in **Figure 2**.

From this figure, we can see that each image is first hand-annotated, and then the corresponding data is exported in ‘JSON’ format for further processing. For each image, one or more masks are created from the exported data, and then they are superimposed to create the binary mask of the image. The images, along with their corresponding masks, are then fed into EmergeNet for training.

### 3.3 Proposed method: EmergeNet

**Figure 3:**
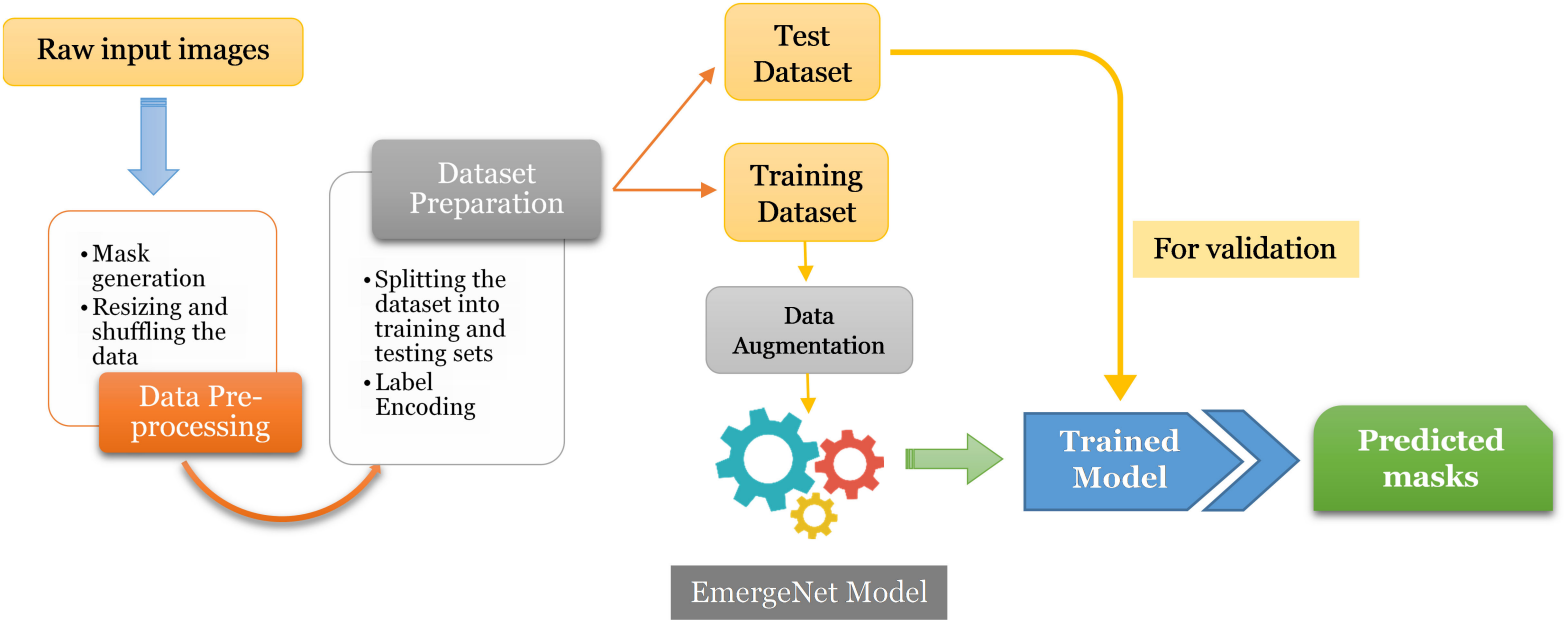
Flowchart of the proposed method.

In this section, we discuss the proposed model and its constituent parts in detail. **Figure 3** represents the block diagram of the proposed method.

The first step is to generate masks from raw input images and pre-process them for training and evaluation. The images and their corresponding pixel labels are partitioned for training and testing. The training dataset is augmented to reduce overfitting and then fed to the model, EmergeNet, for training while the test dataset is used for evaluating the performance of the model. It can then be used to predict the emergence time of an image sequence. EmergeNet is a custom-made ensemble segmentation model that uses a weighted combination of loss functions and is specifically designed to detect tiny coleoptiles at the time of their first emergence from the soil under challenging conditions. It can also be used for growth monitoring of the plant over a time-lapse image sequence. EmergeNet consists of three underlying backbone architectures. The following subsections discuss these three standard backbone models, the EmergeNet Loss function, and the ensembling technique.

#### 3.3.1 The backbone architectures

EmergeNet is built by ensembling three pre-trained networks, each based on the UNet architecture but with modified backbone models. It uses a custom-made loss function as well. The three backbone models used are SEResNet, InceptionV3, and VGG19.

##### 3.3.1.1 The UNet architecture

The UNet architecture, which is an extension of an encoder-decoder convolutional network, is known for its precise segmentations using fewer training images. Therefore, it is only logical to make optimum utilization of the UNet architecture for the task of fine-grain semantic segmentation. The basic intuition behind UNet is to encode the image, passing it through a convolutional neural network as it gets downsampled, and then decode it back, or upsample it to obtain the segmentation mask. However, it is experimentally found that using a pre-trained model as its encoder and decoder, rather than using the standard UNet architecture, the performance of the model improves significantly (Lagree et al., 2021).

##### 3.3.1.2 UNet with SEResNet backbone

A novel architectural unit called the squeeze-and-excitation (SE) block has been introduced in (Hu et al., 2018). It adaptively recalibrates channel-wise feature responses by explicitly modeling interdependencies between channels at almost no computational cost. This is achieved by mapping the input to the feature maps for any given transformation. A detailed description of the structure of SE block and its operational characteristics are provided in (Hu et al., 2018). As an example, adding SE blocks to ResNet50 results in almost the same accuracy as ResNet101, but at a much lower computational complexity.

##### 3.3.1.3 UNet with InceptionV3 backbone

The Inception architecture, unlike conventional convolutional networks, is a very complex, heavily engineered, deep neural network that uses filters of multiple sizes operating at the same level, rather than stacked convolutional layers. It enhances the utilization of available computational resources as well as improves performance significantly. The main idea of the Inception architecture is to find out how an optimal local sparse structure in a convolutional vision network can be approximated and covered by readily available dense components. InceptionV3 makes several improvements over earlier versions by including the following features: (a) *Label smoothing*, which is a regularization technique designed to tackle the problem of overfitting as well as overconfidence in deep neural networks; (b) *Factorizing convolution* to reduce the number of connections/parameters without decreasing the network efficiency; and (c) *Auxiliary classifier* which is used as a regularizer. InceptionV3, with its 42-layer-deep network, is computationally cheaper and much more efficient than other deep neural networks (Szegedy et al., 2016).

##### 3.3.1.4 UNet with VGG19 backbone

The VGG model (Simonyan and Zisserman, 2015) derives inspiration from its predecessor, AlexNet, and is a much-improved version that uses deep convolutional neural layers to achieve better accuracy. VGG19 is the successor to the VGG16 model with 19 layers. VGG19 achieves better accuracy (Shu, 2019) than the VGG16 model as it can extract features better with its deep convoluted network. VGG19 has 16 convolutional layers with 3 FC layers and 5 pooling layers. Here, 2 of the 3 FC layers consist of 4096 channels each. The final FC layer originally had 1000 channels, followed by a SoftMax layer.

We have used the previously discussed three models as the backbone for EmergeNet, replacing the encoder part of the UNet with one of the models at a time. Owing to the symmetric structure of the UNet model, in the decoder or the expansion path, we programmatically upscale the corresponding model in a symmetric fashion to get the final output. For example, if we use VGG19 as the backbone, we are replacing the encoder part of the UNet with the VGG architecture, and in the expansion path, we are using the same VGG architecture to programmatically upscale it. These backbone models were previously trained on the significantly large well-known dataset called ‘ImageNet’, which consists of 3.2 million images (Deng et al., 2009). Thus, using these pre-trained weights allows us to benefit from transfer learning for improved accuracy and speed.

#### 3.3.2 EmergeNet loss function

Instead of the traditional ‘binary cross-entropy’ loss (the negative average of the log of corrected predicted probabilities), EmergeNet uses a weighted sum of the two loss functions which are relevant to the task of segmentation. They are the Dice coefficient loss and focal loss. The motivation for using these two loss functions instead of cross-entropy loss is that these functions address some of the limitations of traditional cross-entropy loss. The statistical distributions of labels play a big role in training accuracy when using cross-entropy loss. The training becomes more difficult as the label distributions become more unbalanced. This is because cross-entropy loss is calculated as the average of per-pixel loss without knowing whether its adjacent pixels are boundaries or not. The Dice coefficient loss and the focal loss, discussed in detail, address these disadvantages and therefore boost the performance of the model.

**Figure 4:**
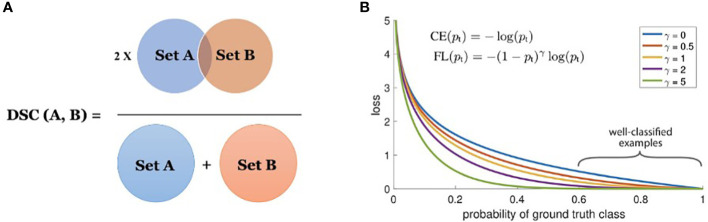
**(A)** Dice coefficient (set view); and **(B)** focal loss for *γ*∈[0,5].

The Dice coefficient is a statistic used to gauge the similarity of two samples and was independently developed by Thorvald Sørensen and Lee Raymond Dice (Sørensen–Dice coefficient, 1948). It was brought to the computer vision community by (Milletari et al., 2016) for 3D medical image segmentation. The Dice loss is computed by


(1) $$D=\frac{2*\sum_{i}^{N}p_{i}*g_{i}}{\sum_{i}^{N}p_{i}^{2}+\sum_{i}^{N}g_{i}^{2}}$$

where, *p_i_* and *g_i_* represent pairs of corresponding pixel values of prediction and ground-truth, respectively. The values of *p_i_* and *g_i_* are either 0 or 1 in boundary detection scenarios, therefore the denominator becomes the sum of the total boundary pixels of both prediction and ground-truth and the numerator becomes the sum of correctly predicted boundary pixels because the sum increments only when *p_i_* and *g_i_* match (both of value 1). **Figure 4A** shows the Venn diagram for the Dice loss.

The Dice similarity coefficient (DSC) is a measure of the overlap between two sets (see **Figure 4A**). In the task of boundary detection, the ground-truth boundary pixels and predicted boundary pixels can be viewed as two sets. By leveraging Dice loss, the two sets are trained to overlap gradually as training progresses. The denominator considers the total number of boundary pixels at a global scale, while the numerator considers the overlap between the two sets at a local scale. Therefore, DSC considers the loss information both locally and globally, making it a very effective loss metric for segmentation.

Focal loss, developed by (Lin et al., 2017), is a modified version of the Cross-Entropy (CE) loss. In the focal loss, the loss for correctly classified labels is scaled down so that the network focuses more on incorrect and low-confidence labels. In the task of segmenting a tiny foreground that relies on pixel-wise classification, a huge class imbalance occurs due to the presence of a considerably large background. Easily classified negatives comprise the majority of the loss and dominate the gradient. The focal loss is designed to address this issue by modifying the CE loss equation. The CE loss for binary classification is given as:


(2) $$CE\left(p,y\right)=\{\begin{array}{ll} -\log\;(p), & \text{if }y=1\\ -\log\;(1-p), & \text{otherwise} \end{array}$$

where *y*∈±1 specifies the ground-truth class and *p*∈[0,1] is the model’s estimated probability for the class with label *y*=1 .

(Lin et al., 2017) proposed to reshape the loss function to down-weigh easy examples and therefore focus more on training on hard negatives. Mathematically, they proposed to add a modulating factor (1−*p*_*t*_)^*γ*^ to the CE loss, with tunable focusing parameter *γ*≥0 . Focal Loss is therefore defined as:


(3) $$FL\left(p_{t}\right)=-\left(1-p_{t}{)}^{\gamma }\log\;(p_{t}\right)$$

where *p_t_* is given by the equation:


(4) $$p_{t}=\{\begin{array}{ll} p, & \text{if }y=1\\ 1-p, & \text{otherwise} \end{array}$$

The focal loss is visualized for several values of *γ*∈[0,5] in **Figure 4B**.

From **Figure 4B**, we note the following properties of the focal loss:

- When an example is misclassified and *p*_*t*_ is small, the modulating factor is near 1 and the loss is unaffected.
- As *p*_*t*_→1 , the factor goes to 0 and the loss for well-classified examples is down-weighted.
- The focusing parameter *γ* , smoothly adjusts the rate at which easy examples are down-weighted.

The EmergeNet Loss function, *loss*_*e*_ , is calculated by the weighted sum of Dice coefficient loss, i.e., *loss*_*d*_ (defined in Eq 1), and focal loss, i.e., *loss*_*f*_ (defined in Eq 3) as follows:


(5) $$loss_{e}=loss_{d}+\alpha \times loss_{f}$$

where *α* is the tuning factor. For our experiment, it has been experimentally found that the optimal value of *α* is 1.

#### 3.3.3 Performance-based weighted ensemble learning

Ensemble learning is a process by which multiple models are strategically generated and combined to solve a particular computational intelligence problem for improved performance. An ensemble model is typically constructed in two steps. First, a number of base learners are built either in parallel or in a sequence. Then, the base learners are combined using popular techniques like majority voting or weighted averaging. There are three main reasons (Dietterich, 1997) why the generalization ability of an ensemble is usually much stronger than that of a single learner:

- The training data might not provide sufficient information for choosing a single best learner. For example, many base learners could perform equally well on the training dataset. Therefore, combining these learners might be a better choice.
- The search processes of the learning algorithms might be imperfect. For example, it might be difficult to achieve a unique best hypothesis, even if one exists, since the algorithms result in a sub-par hypothesis. This can be mitigated by the use of ensemble learning.
- The hypothesis space being searched for might not contain the true target function, while ensembles can give some good approximation.

Instead of using state-of-the-art ensembling techniques like bagging or boosting, EmergeNet introduces a novel weighted ensembling technique that aims to calculate the weights of the individual models based on their performances. These weights are then used to reward or penalize the models. Let *IoU*_*i*_ be the Intersection over Union (IoU) score of the *i^th^* model. We define a penalizing factor *p_i_* as:


(6) $$p_{i}=\frac{{\left(100-IoU_{i}\right)}^{2}}{\sum^{}{\left(100-IoU_{i}\right)}^{2}}$$

The optimal weight, *w_i_* is then calculated as:


(7) $$w_{i}=\frac{\frac{1}{p_{i}^{2}}}{\sum^{}\frac{1}{p_{i}^{2}}}$$

Finally, the ensembled weighted IoU of the EmergeNet (*IoU*_*w*_ ) is computed as follows:


(8) $$IoU_{w}=\sum^{}IoU_{i}\cdot w_{i}$$

**Figure 5:**
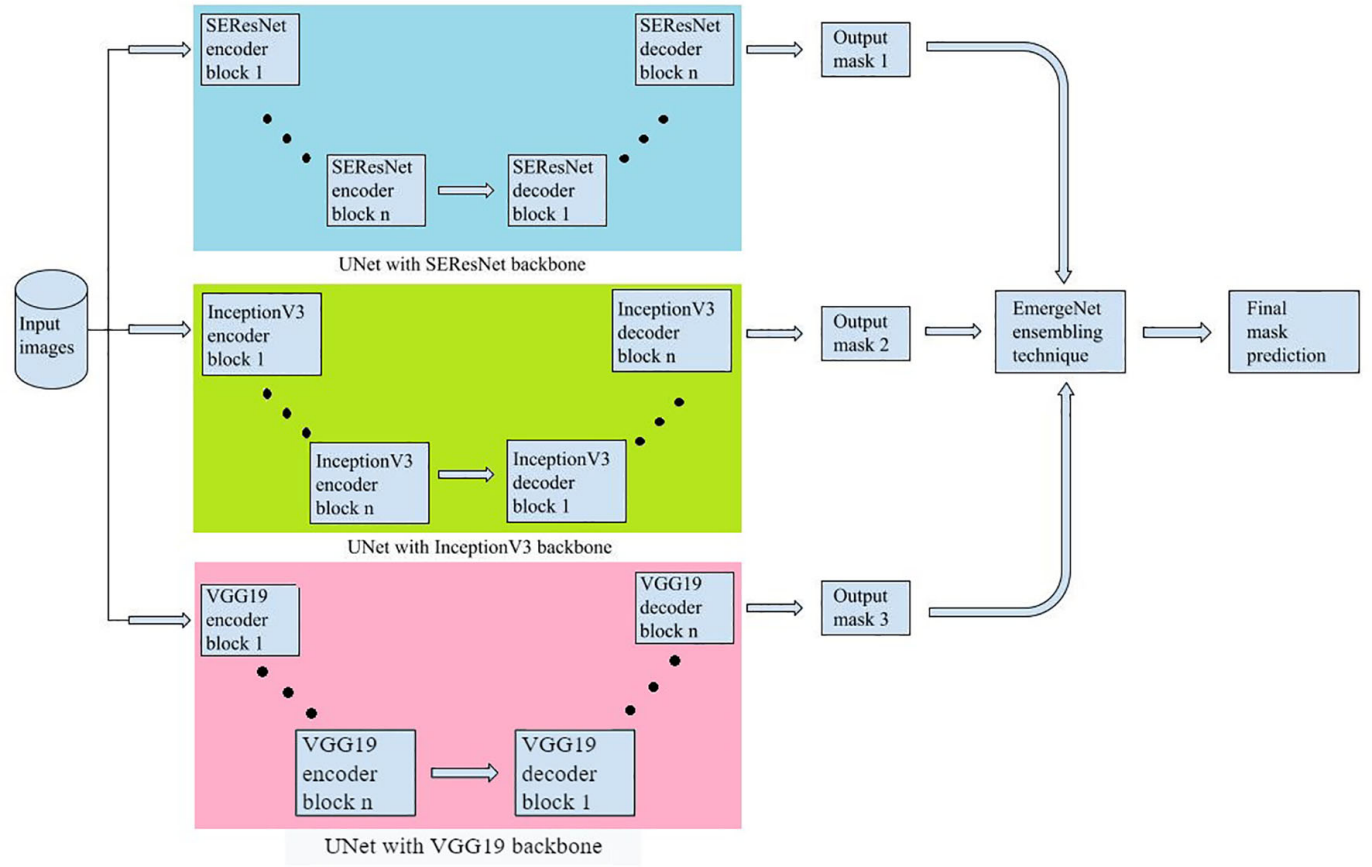
The proposed EmergeNet architecture.

**Figure 5** shows a compact view of the proposed EmergeNet architecture.

### 3.4 Evaluation metrics

The performance of our proposed EmergeNet model has been evaluated using three evaluation metrics, namely, F1-Score, Matthews Correlation Coefficient (MCC) (Matthews, 1975), and Intersection over Union (IoU) whereas the emergence time detection is evaluated using our proposed Emergence Time Accuracy (ETA). Accuracy (or Pixel Accuracy) is not a reliable metric for the task of segmenting tiny objects because this metric is strongly biased by classes that take a large portion of the image. Therefore, we have not used accuracy as a performance metric in this study. It is worth noting that a True Positive (TP) is an outcome where the model correctly predicts the positive class. Similarly, a True Negative (TN) is an outcome where the model correctly predicts the negative class. A False Positive (FP) is an outcome where the model incorrectly predicts the positive class and a False Negative (FN) is an outcome where the model incorrectly predicts the negative class. These metrics are defined as follows:

- F1-Score is the Harmonic Mean between precision and recall. The range for F1-Score is [0, 1], with 0 being the worst and 1 being the best prediction. It is governed by the equation:

(9) $$F1-Score=\frac{2\times TP}{2\times TP+FP+FN}$$

- MCC is an improved metric which takes into account true and false positives and negatives and is generally regarded as a balanced measure that can be used even if the classes are of very different sizes. It has a range of -1 to 1 where -1 is a completely negative correlation between ground-truth and predicted value whereas +1 indicates a completely positive correlation between the ground-truth and predicted value.

(10) $$\text{MCC}=\frac{\text{ TP}\times TN-FP\times FN}{\sqrt{\left(TP+FP\right)\left(TP+FN\right)\left(TN+FP\right)\left(TN+FN\right)}}$$

- Intersection over Union (IoU) is a number from 0 to 1 that specifies the amount of overlap between the prediction and ground-truth.

(11) $$IoU=\frac{Area\;of\;Overlap}{Area\;of\;Union}$$

The emergence time is defined as the timestamp of the image in which EmergeNet first detects the coleoptile(s).

Let *M*={*α*_1_,*α*_2_,…,*α*_*n*_} , where *α_i_* denotes the image for a seeded pot obtained at timestamp *t_i_*, *n* denotes the total number of images in the sequence, where *t*_*i*_ < *t*_*i*+1_ , ∀ 1≤*i*<*n* . The emergence time for a pot is given by the first timestamp EmergeNet finds the coleoptile. Thus,


(12) EmergenceTime(M)=j:EmergeNet(j)≠∅ AND EmergeNet(i)=∅,1≤i<j

The emergence time accuracy (ETA) is determined by comparing the time computed from the results from EmergeNet with the ground-truth, obtained by careful manual inspection of the image sequence.

Given an image sequence, the detection of the emergence time is considered accurate if the time predicted based on the results of EmergeNet (Eq 12) matches the ground-truth, i.e.,


(13) EmergenceTime(M)=GroundTruth(M),

where *GroundTruth*(*M*) is the timestamp of the emergence determined manually.

ETA is given by the proportion of emergences accurately identified by EmergeNet. Thus,


(14) $$ETA=\frac{Number\;of\;emergences\;correctly\;detected\;by\;EmergeNet\times 100\%}{Total\;number\;of\;emergences\;in\;the\;image\;sequences}$$

## 4 Experimental analysis

This section discusses the experimental setup, the benchmark dataset, the evaluation metrics used to evaluate the proposed method, and the results obtained from our experiments.

### 4.1 Experimental setup

The experimental analyses are performed using the Kaggle Notebook, a cloud computational environment that provides a free platform to run code in Python using dedicated GPUs. Kaggle Notebooks run in a remote computational environment and each Notebook editing session is provided with many resources. We used a GPU Kernel with Tesla P100 16 GB VRAM as GPU, with 13 GB RAM along with a 2-core of Intel Xeon as CPU. The training masks are generated using the VGG annotator tool. Python is featured with a plethora of useful packages, like, OpenCV, TensorFlow, Keras, Scikit-learn, etc., which are used to train the model and evaluate its performance. The number of images used for training was 260 (130 images and their corresponding masks). The execution time for training the EmergeNet was 1.5 hours. Compared to other deep neural networks, EmergeNet took less time to train as it benefits from transfer learning. We trained each model until the IoU curve for each of them reached saturation, and no further improvement was possible.

### 4.2 Results

We present our results in two different parts. First, we present a comparative study of EmergeNet and UNet in terms of performance. We have also compared the performance of all three individual backbone networks with EmergeNet. A more detailed analysis of the performance of EmergeNet under dark lighting conditions is also presented. In the second part, we analyzed the growth monitoring of maize coleoptiles as well as their emergence timing detection.

#### 4.2.1 Comparative study

**Figure 6:**
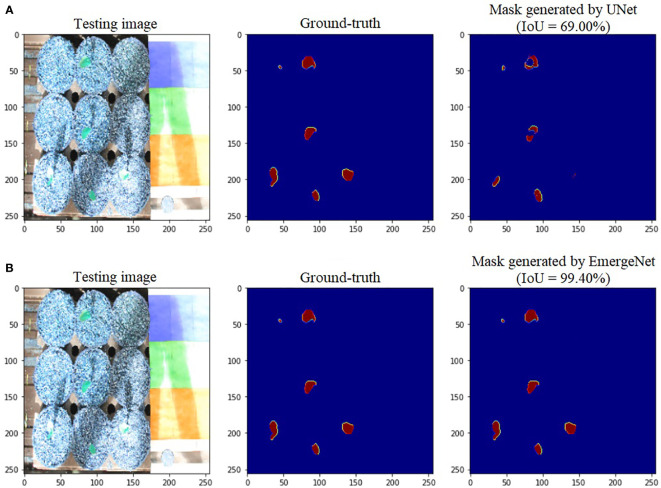
Illustration of segmentation performance on a test image from UNL-MED by **(A)** UNet and **(B)** EmergeNet.

**Figure 6** shows the comparison of masks generated by UNet and EmergeNet using a test image sequence from the UNL-MED. **Figure 6A** shows the masks generated by the standard UNet model and their corresponding ground-truth. Note that the generated masks do not accurately match with the ground-truth. Furthermore, the UNet model failed to detect the emergence of coleoptiles in several cases. This is generally the case with generic models which are not sophisticated enough. The IoU obtained by the standard UNet model for this test sample is 69%. **Figure 6B** shows the masks generated by the proposed EmergeNet model and their corresponding ground-truth.

In contrast to the UNet model’s results, the masks generated using EmergeNet closely correspond to the ground-truth. EmergeNet neither incorrectly generated a mask (when there was no emergence), nor failed to produce a mask when there was a coleoptile. The overall IoU of EmergeNet for this test sample is 99.40%, a significant improvement over UNet. **Table 1** shows the comparative analysis of UNet and EmergeNet in terms of the three evaluation metrics, namely, F1-Score, MCC, and IoU, for all images of UNL-MED. It is evident from the table that EmergeNet significantly outperforms the UNet model.

**Table 1:** Results of comparison between UNet and EmergeNet on UNL-MED under all lighting conditions in terms of F1-Score, MCC, and IoU.

	F1-Score	MCC	IoU
UNet	0.6813	0.7040	0.7690
EmergeNet	0.9470	0.9894	0.9820

**Figure 7:**
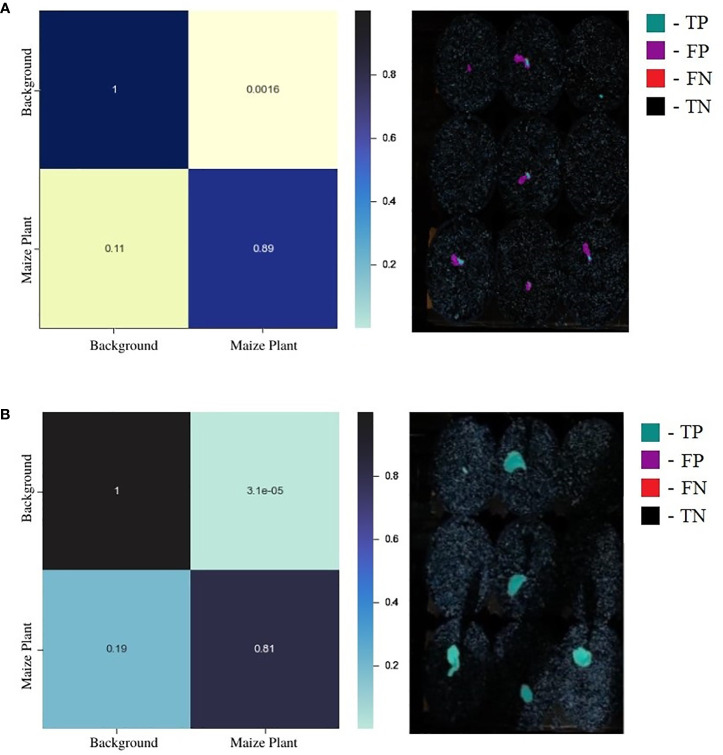
**(A)** Juxtaposition of confusion matrix (left) and its corresponding overlay mask from standard UNet (right). **(B)** Juxtaposition of confusion matrix (left) and its corresponding overlay mask from the EmergeNet (right).

While a confusion matrix (CM) is a powerful visualization technique to summarize the performance of a supervised classification task, it does not provide valuable insights into the model’s performance for image segmentation since the data is highly imbalanced toward the background class. The normalized CMs for a random test image for the standard UNet and EmergeNet are shown in **Figure 7**. It is evident from the figure that the background is significantly larger than the maize coleoptile. A more accurate representation of the classifier’s performance can be derived by overlaying the values of the confusion matrix on the coleoptile mask generated by the classifier. **Figure 7A** shows that majority of the mask generated by the standard UNet (shown in magenta) is incorrectly labeled as the coleoptile, i.e., false positive. Only a very small portion of the mask is accurately labeled (shown in cyan), denoting the true positives. **Figure 7B** shows the mask overlaid by the values of the corresponding confusion matrix for EmergeNet. It shows that a significant majority of the mask is correctly labeled (shown in cyan), and only a few pixels, mostly along the border, are false positives (shown in magenta). There are no false negatives or true negatives for EmergeNet. This demonstrates the efficacy of EmergeNet and its superiority over the standard UNet for this application.

**Figure 8:**
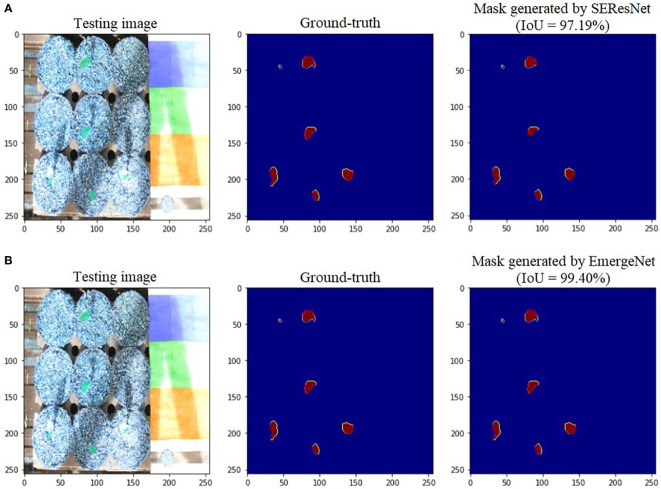
Illustration of segmentation performance on a test image from UNL-MED by **(A)** SEResNet and **(B)** EmergeNet.

**Figure 9:**
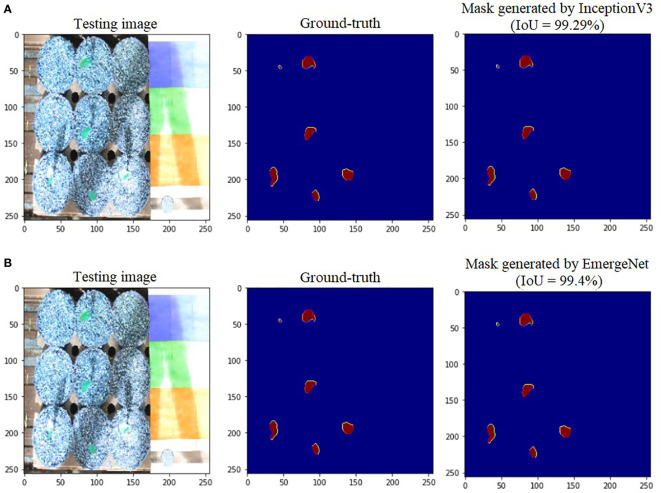
Illustration of segmentation performance on a test image from UNL-MED by **(A)** InceptionV3 and **(B)** EmergeNet.

**Figure 10:**
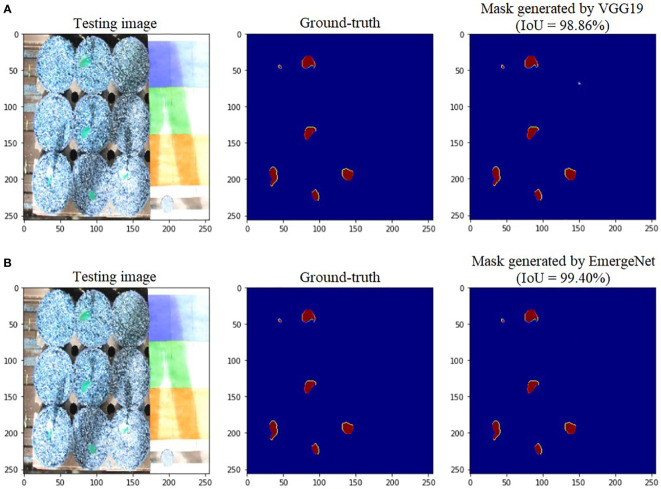
Illustration of segmentation performance on a test image from UNL-MED by **(A)** VGG19 and **(B)** EmergeNet.

To further add credibility to the accuracy and robustness of the integrated network, i.e., EmergeNet, we performed a comparative study among the individual networks with EmergeNet. In each case, the masks generated by EmergeNet are better than that created by the individual networks. **Figure 8** compares the masks generated by UNet with SEResNet as its backbone, and EmergeNet. The IoU of EmergeNet is higher by 2.21%. InceptionV3 is a very powerful network on its own, and therefore, the UNet structure with InceptionV3 as its backbone is expected to perform remarkably well. Such is the case as depicted in **Figure 9**, however, EmergeNet still beats the IoU score by 0.11% which is impressive considering the fact that it becomes exponentially more difficult to improve the results above a certain threshold value. Finally, EmergeNet beats UNet with VGG as its backbone by 0.54% in terms of IoU metrics as shown in **Figure 10**. Conclusively, it can be inferred that the integrated structure of EmergeNet plays a significant role in bringing the best of all the individual networks and performs better than all of them, thereby proving its worth as a segmentation model.

To demonstrate the efficacy of EmergeNet under extremely low light conditions, we conducted the same experimental analyses by considering the images of UNL-MED that were captured in a dark environment only. **Table 2** summarizes the results of the comparison between the UNet and EmergeNet in dark conditions. Results show that EmergeNet significantly outperformed the standard UNet along all three evaluation metrics.

**Table 2:** Results of comparison between UNet and EmergeNet on UNL-MED only under dark environmental conditions in terms of F1-Score, MCC, and IoU.

	F1-Score	MCC	IoU
UNet	0.6065	0.6000	0.7774
EmergeNet	0.9709	0.9709	0.9735

#### 4.2.2 Growth monitoring

**Figure 11:**
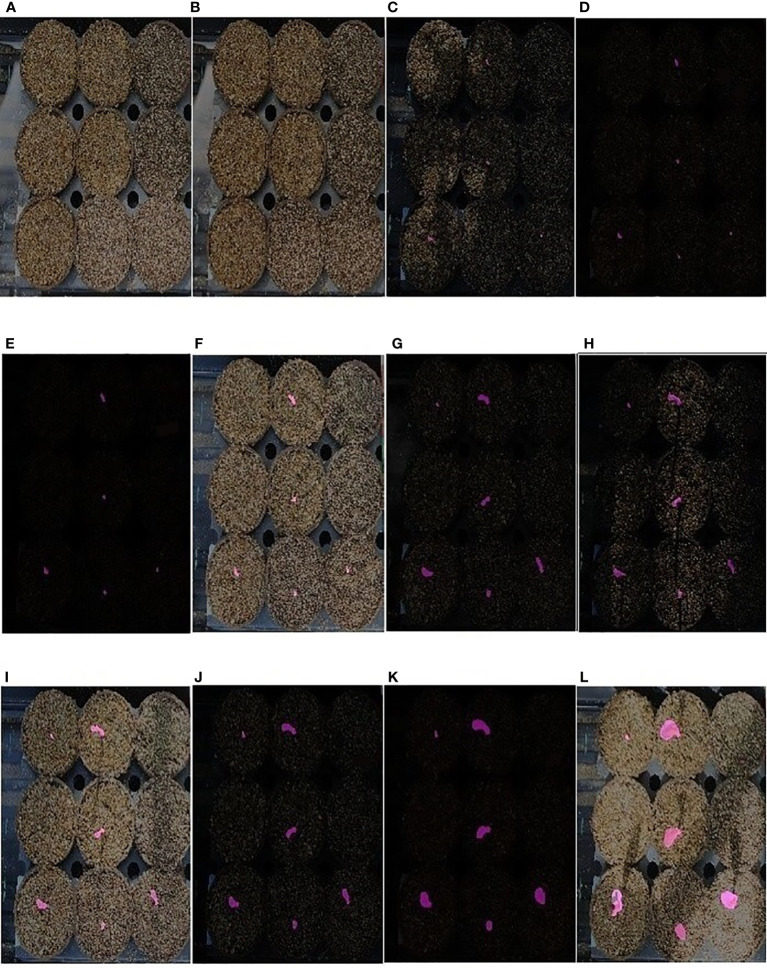
**(A–L)** Illustration of emergence timing detection and growth monitoring of a maize coleoptile in a time-lapse test image sequence of UNL-MED.

Thus, EmergeNet can efficiently monitor the growth of maize coleoptiles even at extremely low light conditions. We defined a new measure called the ETA to evaluate the accuracy of emergence in Section 4.4. **Figure 11** displays a sequence of images that show the emergence and growth of coleoptiles computed by EmergeNet. It shows that EmergeNet detected the emergence of coleoptile with 100% ETA. Furthermore, it correctly tracks the growth of the coleoptile, as demonstrated by the increasing size of the generated masks in the time-lapse imagery. Note that the first two subfigures, i.e., **Figures 11A, B**, do not contain any masks because the emergence has not taken place yet. Out of nine maize seeds sown in the nine pots, only four of them emerged earlier, as shown by the tiny masks in **Figure 11C**. Subsequently two more coleoptiles emerged, one in **Figure 11D** and the other in **Figure 11G**. Three seeds failed to emerge in the experiment. After their emergence, all six coleoptiles are correctly tracked. Thus, EmergeNet accurately identifies the emergence events and tracks the growth of the coleoptiles over time.

**Figure 12:**
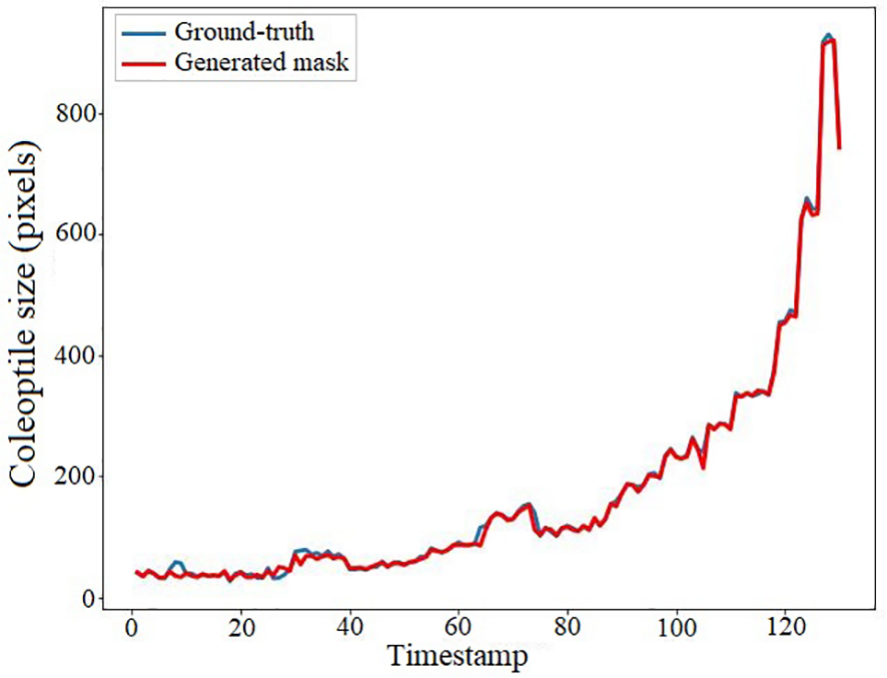
Coleoptile size measured by the total number of constituent pixels of the ground-truth (shown in blue) and the generated mask (shown in red) as a function of time.

We conducted an experiment to demonstrate the accuracy of the coleoptile size measured by the total number of constituent pixels of the generated masks by comparing them with the ground-truth. The result of the comparison is shown in **Figure 12**. The figure shows the coleoptile size of the generated mask (shown in blue) significantly overlaps with the coleoptile size of the ground-truth (shown in red). Thus, EmergeNet not only produces high-precision masks but also helps in the growth monitoring of coleoptiles with very high accuracy. **Table 3** shows the Pearson correlation coefficient between the coleoptile size of the mask generated by EmergeNet and the ground-truth. Pearson correlation coefficient is a measure of the linear relationship between two variables. It has a value between -1 to 1, with a value of -1 denoting a total negative linear correlation, 0 being no correlation, and + 1 denoting a total positive correlation. The table shows a high positive correlation between the ground-truth mask and the generated mask in terms of coleoptile size.

**Table 3:** Pearson correlation table between ground-truth and generated mask.

	Ground-truth	Generated mask
Ground-truth	1.000000	0.999412
Generated mask	0.999412	1.000000

## 5 Discussion

The timing of germination is a paramount physiological factor for seed quality determination that encompasses a set of broad concerns, including vigor, dormancy mechanisms, pests, pathogens, genetic integrity, cost of establishment, field maintenance to prevent contamination with weeds or unwanted seed, and isolation distances to prevent cross-pollination Stiller et al. (2010). Thus, research attention for automated emergence timing determination based on computer vision and artificial intelligence techniques to replace tedious manual human labor is more crucial than ever. In this paper, we proposed an ensemble deep-learning based segmentation model based on the UNet architecture for coleoptile emergence time detection. The proposed EmergeNet outperforms the UNet by a significant margin as demonstrated by the experimental analyses in Section 4.2. The success of EmergeNet is attributed to successful base backbone architectures, customized loss function, and a novel penalizing factor in the ensemble technique. The accuracy of any image-based phenotypes depends on the accuracy of the underlying segmentation model Das Choudhury (2020). Thus, the ensemble segmentation model introduced in this paper has the potential to be extended to other automated phenotyping applications.

The proposed EmergeNet model significantly outperforms the widely used UNet architecture. The IoU metric for mask generation for EmergeNet is 99.4%; in comparison, the standard UNet has an IoU of 69% (see **Table 1**). By combining the three UNet architectures with powerful backbones, along with the proposed custom loss function and a novel ensemble technique, EmergeNet reduces the number of false positives (see **Figure 7B**). One of the key contributions of this model is its success in extremely low light, as evident from **Table 2**. Overall, EmergeNet is able to detect the emergence timing of the maize coleoptile with 100% accuracy. Furthermore, EmergeNet can accurately monitor the growth of the coleoptiles over time, as demonstrated by a very high correlation (0.999) between the generated masks and the ground-truth.

## 6 Conclusion

The timing of important events in a plant’s life, for instance, germination, the emergence of a new leaf, flowering, fruiting, and onset of senescence, is crucial in the understanding of the overall plant’s vigor, which is likely to vary with the interaction between genotype and environment, and are referred to as event-based phenotypes. This paper introduces a novel deep-learning model called EmergeNet to detect the timing of the emergence of a maize seedling and track its growth over a time-lapse video sequence. EmergeNet is based on an ensemble model that integrates SEResNet18, InceptionV3, and VGG19, such that it overcomes the challenge of detecting a tiny living object and tracks its changes in shape and appearance in the presence of cluttered soil background and extreme variation of illuminations. Furthermore, the paper introduces a benchmark dataset called UNL-MED. Experimental evaluation on UNL-MED shows the capability of EmergeNet to detect the timing of emergence with 100% accuracy as compared with human-perceived ground-truth. It is also experimentally demonstrated that EmergeNet significantly outperforms its base model UNet in the task of segmentation. EmergeNet incorporates three pre-trained networks including all their weights, and hence, it requires high-end computing power for efficient training. Additionally, EmergeNet is trained on only one type of plant constrained by a set of external environmental conditions. Future work will consider the detection of multiple coleoptiles with or without the presence of weeds in the same pot.

## Data availability statement

The datasets presented in this study can be found in online repositories. The names of the repository/repositories and accession number(s) can be found below: https://plantvision.unl.edu/dataset.

## Author contributions

AD developed and implemented the algorithm, conducted experimental analysis, and contributed to manuscript writing. SD conceived the idea, led the dataset design, conducted experimental analysis, led the manuscript writing, and supervised the research. AS, AKD, and TA critically reviewed the manuscript and provided constructive feedback throughout the process. All authors contributed to the article and approved the submitted version.
